# Radiology reporting—from Hemingway to HAL?

**DOI:** 10.1007/s13244-018-0596-3

**Published:** 2018-03-14

**Authors:** Adrian P. Brady

**Affiliations:** Radiology Department, Mercy University Hospital, Grenville Place, Cork, T12 WE28 Ireland

**Keywords:** Image interpretation, computer-assisted [E01.158.600], Quality assurance, health care [N05.700], Diagnostic imaging [E01.370.350], Observer variation [E01.354.753], Clinical decision-making [E01.055]

## Abstract

**Abstract:**

The job of the diagnostic radiologist is two-fold: identifying and interpreting the information available from diagnostic imaging studies and communicating that interpretation meaningfully to the referring clinician. However skilled our interpretive abilities, our patients are not well served if we fail to convey our conclusions effectively. Despite the central importance of communication skills to the work of radiologists, trainees rarely receive significant formal training in reporting skills, and much of the training given simply reflects the trainer’s personal preferences. Studies have shown a preference among referrers for reports in a structured form, with findings given in a standard manner, followed by a conclusion. The technical competence to incorporate structured report templates into PACS/RIS systems is growing, "...and radiology societies (including the European Society of Radiology (ESR)) are active in producing and validating templates for a wide range of modalities and clinical circumstances. While some radiologists may prefer prose format reports, and much literature has been produced addressing “dos and don’ts” for such prose reports, it seems likely that structured reporting will become the norm in the near future. Benefits will include homogenisation and standardisation of reports, certainty that significant information has not been omitted, and capacity for data-mining of structured reports for research and teaching purposes.

****Teaching Points**:**

*• The radiologist’s job includes interpretation of imaging studies AND communication.*

*• Traditionally, communication has taken the form of a prose report.*

*• Referrers have been shown to prefer reports in a structured format.*

*• Structured reports have many advantages over traditional prose reports.*

*• It is likely that structured reports represent the future standard.*

## Introduction

Glendower: *I can call spirits from the vasty deep.*

Hotspur: *Why so can I, or so can any man,*

*But will they come when you do call for them?*

(William Shakespeare, Henry lV, Part 1, Act 3, Scene 1)

Every radiologist knows (or thinks they know) how to report; it is what we do all day. But how many of us have thought about the process and details of our efforts to communicate and their effectiveness? Having, we think, called the spirits, have they come?

Many of us pride ourselves on our diagnostic acumen, our ability to tease clues from the vast amount of visual data presented to us and to arrive at a confident diagnosis. We believe these skills are key to our role in patient care and that making correct diagnoses from imaging data constitutes the essence of doing our job well. But this is only part of our responsibility. Diagnostic accuracy is useless if the conclusions arrived at are not communicated effectively to the referrer. If we fail to pass on our understanding of the meaning of imaging findings, or if the referrer does not appreciate what we mean in our reports, we have failed in our duty to our patients. Our job has two key elements: correct interpretation of imaging information and effective communication of that interpretation. I fear we succeed more commonly in the first element than in the second.

The essential work product of the diagnostic radiologist is the radiology report. Report production is not always an exact science; it has been described as “the art of applying scientific knowledge and understanding to a palette of greys, trying to winnow the relevant and important from the insignificant, seeking to ensure the word-picture we create coheres to a clear and accurate whole, and aiming to be careful advisors regarding appropriate next steps”. We may try to be the best “sifters of information and artists of communication” we can be, but inevitably these are imperfect processes [1].

## What is a radiology report?

A radiology report represents the culmination of the process of interpreting a radiological study (or detailing what happened during an intervention). It is a formal document, medicolegally important, committing the radiologist to an official interpretation of a single examination or procedure [2]. One of the earliest known extant examples of a radiology report, as we understand the term, is a handwritten letter from Dr. William J. Morton of New York, dated May 1896, describing to a colleague the findings on an abdominal radiograph and mentioning all visible skeletal structures and the absence of renal calculi [3]. Its structure and content are little different from prose reports today.

As early as 1899, Dr. Preston Hickey, a radiologist in Michigan, advocated that reporting of radiographs (indeed, Hickey coined the use of the terms *radiograph* and *interpretation* in the context of our profession) should follow a standardised format and language [2, 3]. In 1922, he suggested that the American Roentgen Ray Society should require all membership applicants to submit 100 radiology reports for assessment; only if the clarity and diagnostic value of the reports were considered adequate should membership be granted [4].

Even in the early days of radiology, the lack of clinical value of poor reports was recognised as a detriment to patients and to the specialty of radiology. In 1923, Charles Enfield, a Kentucky radiologist, wrote in the Journal of the American Medical Association that a radiology report that does not state what the findings mean “tells much, yet almost nothing” and that the ideal report “commits the roentgenologist to his opinion” [4].

However, radiologists’ reports often cannot be definitive or beyond question. In most circumstances, our reports represent clinical consultations, giving our specialist opinion (“a view held about a particular subject or point; a judgement formed; a belief” [5]) on the basis of the information available at the time (which may be limited or incomplete), having weighed the available evidence [6]. Radiological studies do not come with in-built labels denoting the most-significant abnormalities, and interpretation of them is rarely a binary process (normal vs. abnormal, cancer vs. “all clear”) [1]. Certainly, some reports are definitive (often those attached to less-complex studies), but many are better regarded as an invitation to a conversation in the light of any available additional information or the progress of the patient’s clinical condition. Multidisciplinary team meetings provide the opportunity for this conversation [7]. In the light of further information, opinions can change; radiology reports are often not the final word. The public and the media (and sometimes our referring colleagues) do not always understand this and may view a report that ultimately proves incorrect or incomplete as a failure on the part of the radiologist. These “failures” are easily identified and reviewed; almost all our clinical work is archived and available for later re-interpretation [1].

## Recommendations for good reports

Prior to the wide availability of PACS/RIS systems and voice-recognition dictation, the general thrust of literature about radiology reporting focused on the style and content of prose reports. As report generation has become more computer-based, attention has moved to structured reporting, which will be discussed below. Earlier recommendations regarding the advisability of including demographic data about the patient, referrer and investigation in the body of the report [4] have been largely superseded by PACS/RIS systems that automatically record such details.

Many authors have written about what constitutes a “good” report; many of their suggestions have been based as much on personal preference as on any objective standard. In 1983, Friedman wrote: *“It is certainly not necessary for the trained radiologist to use the crutch of reciting regional anatomy as a reminder to look at all parts of the film, and I suspect that it is a bad habit for the trainee to become accustomed to the mindless litany which is often truly lip service, unaccompanied by appropriate eye movements”* and that *“computerized reports [are] no easier read by the clinician if filled with trivia, although they may be more quickly put together by the radiologist”* [8]. Those views are unlikely to find a sympathetic hearing among today’s champions of structured reporting.

If we consider prose-style reports alone, there is no consensus about what constitutes a good report [9]. Some advocate for well-constructed complete sentences [10, 11], following a hierarchy of terms, with a rich, precise vocabulary [12]. Others believe that complete sentences, while nice if the radiologist can use them gracefully, are unnecessary for effective communication of information [9, 13].

Many have suggested that report length often is in inverse proportion to the confidence of the radiologist in his conclusions [8, 9]: “*During my radiology residency training (1985-1989), I was instructed that, as a radiologist, I was paid for both my eyes and my brain and that a complete radiology report had to include both sets of evaluations….[t]he length of the body of the report depended on the number of findings, whereas the length of the conclusion varied with my ability to make sense of the findings*” [14].

Many papers have been published detailing words and forms of language that should or should not be used in radiology reports (with subsequent discussions about the relative merits and demerits of the specific suggestions in the journals’ letters pages) [2, 8–12, 14–20]. I do not propose to repeat the “dos and don’ts” of these publications, many of which are worthy of individual review for their common sense and elucidation of the pitfalls of the careless use of imprecise language. Armas summarised the qualities of a good report as the six Cs:

1. Clear
2. Correct
3. Confidence level, which should be indicated
4. Concise
5. Complete*(“some radiologists’ definition of complete includes a detailed description of everything short of the weather report”)*
6. Consistent

All of which come together as the seventh “C”: communication [21].

## Hedging

*“[M]aster radiologist…has once again demonstrated his uncanny ability to hedge on every conceivable diagnosis when reviewing an imaging study…has successfully raised all known disorders as possible aetiologies of the findings visible on the scan….concludes his diagnosis with his signature signoff: ‘Clinical correlation required’”* [22].

*“Radiology intern…stunned colleagues and associates with his record-setting hedge. [C]olleagues were quick to congratulate him on such an impressive feat of hedging. ‘I was in practice for two or three years before I was that good at avoiding diagnostic commitment’, said MSK radiologist Chuck Wilson. ‘Heck, I’m not even sure I’m that good now’”* [23].

If these humorous web articles do not make you a little uncomfortable, then you are either an exceptionally disciplined radiologist of consistent clarity and certainty or you are in denial. Almost all of us step back from commitment in our reports on occasion. Some of us do it much more frequently.

On other occasions, we (consciously or otherwise) insert language in our reports that diminishes the certainty (and therefore often the usefulness) of the report. Examples include: *“no evidence of…”* [8] and *“apparent, appears, possible, borderline, doubtful, suspected, indeterminate…suggested, suspected, suspicious for, vague, equivocal, no definite, no gross, no obvious”* [9]. The use of the term *“identified”*, as in, for example, *“no fracture identified”*, could suggest a fracture may be present, but we did not invest much effort in looking for one [2].

Sometimes uncertainty is unavoidable. One author suggests that, in such circumstances, the phrases *“almost certainly present”* or *“almost certainly absent”* may be useful if we think it advisable for the patient’s sake to stop further imaging workup [3]. Others propose using the first person (as in *“I am unsure as to the significance of this”)* to add a personal touch when equivocation is unavoidable [2].

All too often, we hedge. The hedge has been described as the “tree of our specialty” [2]; it has been suggested that the radiology logo should *“depict a weasel eating a waffle under a hedge”* [3]. No test has an accuracy rate of 100%, and absolute certainty in radiology is rare, but we should not hide behind this fact to cultivate the semblance of never being wrong (by never reporting anything definite). Consistently hedged reports are useless and are seen as such by referring clinicians, with consequent loss of respect for our specialty. *“Get off the fence or explain why you are on it”* [13].

Hedging can apply to reluctance by a radiologist to use the term “normal”. The confident use of “normal” in a report can have great value in ruling out disease and should be encouraged. Yet all too often we shy away from this unequivocal word and replace it with what Langlotz has described as “normal imposters”, inherently much less certain. Among these, he lists:

- *“Unremarkable”*—then why are you remarking on it? It means “lacking distinction, ordinary”
- *“Grossly normal”, “essentially normal”*—in what nonessential way is it not normal?
- *“Relatively normal”*—relative to what?
- *“No radiographically visible signs of disease”*—a hedge against abnormalities that are not detectable with imaging; any referrer should know the capabilities of radiography.
- *“No significant abnormalities”, “normal for age”*—we should only use these if clinically relevant, e.g. if insignificant or age-related observations are mentioned in the body of the report.

“Limit” describes “a boundary surrounding a specific area”. *“Within normal limits”* should only be used when adhering to that definition, not as a synonym for “normal”. Describing a boundary only makes sense when observing a structure whose internal architecture cannot be discerned, e.g. *“The heart and mediastinum are within normal limits”* on a CXR [3].

A special word about a phrase for which a particular disdain is reserved by many of our clinical colleagues: *“Clinical correlation recommended (CCR)”*. Langlotz has asked how we, as radiologists, would feel if a request for an imaging study contained the instruction *“Recommend examining the images carefully”;* “CCR” may be just as offensive to referring clinicians [3]. Lee and Whitehead surveyed radiologists and non-radiologist clinicians about their understanding of the meaning of a range of phrases commonly used in radiology reports. Both groups interpreted “CCR” as meaning narrowly that *“imaging findings should be interpreted in the context of the clinical picture”*. However, 28% of clinicians and 26% of radiologists thought it meaningless, hedging or an attempt to shift responsibility to clinicians. The authors describe “CCR” as one of the most contentious phrases to appear in radiology reports and suggest its elimination [17].

## What the referrer (and the radiologist) needs and wants

A clinician referring a patient for an imaging study is usually looking for a series of specific things in the radiologist’s report: complete and accurate identification of relevant findings, a coherent opinion about the likely underlying cause of abnormalities and, if appropriate, guidance on further investigations that may add information or certainty. The response of reporting radiologists to these implied requests can range across a wide spectrum, from those who believe it is best to produce a long list of positive and negative findings, and an exhaustive differential diagnosis, through those radiologists who try to achieve a brief but accurate report giving only those findings and differential diagnoses they believe likely, to those whose reports consist of lists of findings without context or filtration. If there is mutual understanding between referrer and radiologist, many of the report variants along this spectrum can achieve the necessary result, but this depends on experience and trust between the referrer and reporter [1].

In the current model of radiology service provision, where contact between referrer and radiologist is diminishing [24, 25], and increased use of off-site reporting may mean the two are completely unknown to one another, it is often impossible for this trust and experience to develop. Referrer A may have no idea what subtle point Radiologist B is trying to convey by the use of particular language. In an increasingly globalised environment, standardisation of language and reporting acquires greater importance.

Clarity of reports is key to accurate communication of meaning. Report readers are usually in a hurry [3]. Referrers often complain about the failure of a radiologist to commit to a conclusion. Rambling descriptions without a useful conclusion add little to patient care and often suggest the radiologist wishes to remain remote from the clinical problem. If we compose unnecessarily vague or ambiguous reports, we do a disservice to our patients and to ourselves [26].

Attempts have been made to identify precisely what types of reports are desired by referrers. In 1995, McLoughlin et al. surveyed 100 referring doctors regarding their preferences among three different styles of report for each of six clinical scenarios. For a normal CXR, a report simply stating *“normal”* was most popular if the patient had no chest symptoms, but if symptoms were present, reports giving descriptive detail were preferred. In abnormal CXRs, most wanted reports describing the findings and suggesting the diagnosis, as opposed to only giving a diagnosis. For abdominal ultrasound studies, most preferred reports giving detailed findings, even if those findings were normal. Thus, the descriptive detail expected by clinicians depended on the clinical circumstances, but was independent of the specialty, experience or academic status of the referrer. The authors speculated that the preference of a substantial proportion of physicians for detailed descriptions, even when this involved listing negative findings, providing no further information, might indicate that referrers interpreted these reports as showing that a thorough examination was performed [27].

In 2005, Sistrom and Honeyman-Buck attempted to identify whether the format of a radiology report (independent of its content) had any effect on the ability of a reader to extract information of relevance to patient care. The working hypothesis was that consistently formatted (structured) reports would be easier to read and understand, with improved efficiency in answering content-specific questions. Sixteen senior medical students were given radiology reports to read in free text or structured format and asked to answer multiple-choice questions about specific medical content. While the subjects all strongly preferred structured format reports to free text, no significant differences were found between the two formats in terms of speed of reading the reports, accuracy of understanding of their content or efficiency of assimilation of the contained information. The authors suggested that overly structured data can result in a loss of cognitive focus by clinicians, with a loss of overview when dealing with data in many fields, and that this can apply to the reporting radiologist as well as the report reader. The act of composing a report in narrative form may be an inherent part of the process of cognitive processing of the case for the radiologist. Nonetheless, they concluded in favour of report organisation and format like a ‘laboratory report’, principally to meet the wishes of the referrer [28].

More recently, at ECR 2017, findings were presented that contrasted with these results. The authors created unstructured reports for CT angiographic studies and CTs of abdomen and structured reports for MRI brain and thoracic CT studies. An online survey of almost 150 clinicians asked subjects to read each report and then asked multiple-choice questions based on the reports (while being unable to return to the report). Critical findings were missed in 34.9% of unstructured reports and 17.3% of structured reports. The incorrect diagnosis was selected by the subjects in 18.1% of unstructured reports and 6.2% of structured reports. Overall, structured reports led to better recall of all (and critical) findings and fewer incorrect diagnoses. The worst-performing report was an unstructured CT coronary angiogram; the authors stated: *“It seems the more complex the study, the greater benefit that you can yield from having a structured report”* [29].

In a 2009 survey of hospital clinicians’ preferences, Plumb et al. found that only 31% of (non-radiologist) consultants believed *“normal examination”* was a sufficient report. (A 2010 survey of GPs’ preferences made a similar finding—respondents did not know what organs had been examined [30]). A majority felt that it was appropriate to include some information regarding examination technique and quality. Recommendations for further investigations were welcomed, and 63% agreed that if further imaging was recommended by a radiologist, it should be automatically arranged. Strong preferences were expressed for more detailed (as opposed to simpler) reports and for tabular reports rather than prose; most preferred was a detailed tabular report accompanied by a radiologist’s comment [31].

In 2011, Bosmans and colleagues surveyed clinical specialists and GPs (COVER) and radiologists (ROVER) regarding their views of and preferences for radiology reports. Eighty-seven per cent of referrers considered the radiology report an indispensable tool; 63% did not think they were better able to interpret an imaging study in their own specialty than a radiologist. Almost all agreed they need to provide adequate clinical information and state clearly the question they want answered. For complex examinations, 84.5% of referring clinicians and 55.3% of radiologists preferred itemised reports; 56% of clinicians and 72.9% of radiologists rejected the idea that a radiology report should consist of prose. Half of referring clinicians thought the radiologist might not have looked at a particular feature if it was not explicitly mentioned in the report; slightly over half of radiologists agreed that clinicians would assume this. The authors concluded that there is no universal consensus on what constitutes a good report and that the literature on this topic is primarily based on the insights and lifetime experience of specific authors rather than formal assessment of the views and needs of referrers or radiologists. Commenting that *“one size does not fit all”*, they recommend tailoring the report to the profile of the referring physician. Finally, they encapsulate the dilemma by noting: *“Medicine certainly needs talented and competent radiologists. But do we want them to be data entry clerks rather than journalists, poets or essayists?”* [32].

Clinicians based in hospitals are able to attend in-house meetings and conferences, interact on a face-to-face basis with radiologists, view images and engage in discussion with the reporting radiologist. Primary care practitioners usually lack most or all of these opportunities and must rely more completely on the content and recommendations in the radiologists’ reports [2, 30]. Furthermore, terminology or concepts familiar to a specialist may be unusual to a primary care physician [2]. For example, the normal range of renal size measurements on ultrasound is likely to be known to a nephrologist; whether a specific measurement is normal or not may need to be specified to a family practitioner. GPs have also been shown not to value inclusion of examination technique or details of contrast media used [30]. It is important that reporting radiologists take into account the likely reader of a report in framing a report; we should try to put ourselves in the position of the likely reader when deciding what to dictate, and how to structure that dictation, ensuring that the reader will understand clearly what we mean to convey.

The list of differential diagnoses we offer may also need to be tailored to the referrer. GPs may prefer a longer differential list than hospital-based specialists (without rambling, suggesting uncertainty) and have been shown to prefer more recommendations about further investigation (radiological and other) [2]. Balance is required to avoid a long list of (perhaps irrelevant) possibilities, diluting the significance of important observations. *“Irrelevant observations have a cost, paid by the distraction they cause from the salient information”* [3]. In addition, recommendations for further investigation must be pertinent and proportionate and not force the hand of a referrer to initiate unnecessary investigation or follow-up [3, 9].

One final point worth bearing in mind is a stated preference of GPs for a high level of detail in reports to facilitate showing the reports to patients [30]. In the current era of easy sharing of information, we must not lose sight of the likelihood that patients will read reports we generate; frivolity or unguarded inappropriate language must be avoided.

## Clinical information

Inadequate clinical information or inappropriate expectations of the capabilities of a radiological technique can lead to misunderstanding or mis-communication between the referrer and the radiologist [1, 33]. (The impact of lack of clinical information may be overestimated, however. In 1997, Tudor evaluated the impact of the availability of clinical information on error rates when reporting plain radiographs. Five experienced radiologists reported a mix of validated normal and abnormal studies 5 months apart, with no clinical information on the first occasion and with relevant clinical information on the second occasion. Mean accuracy improved from 77% without clinical information to 80% on provision of the clinical information, with modest improvements also in sensitivity, specificity and inter-observer agreement [34].)

Nonetheless, the principle is sound: *“rubbish in, rubbish out”*. If radiology reports are to be of value clinically, they must be supported by all relevant clinical information available at the time of the examination.

## Communication

There are two major components to a radiology report. One is the interpretation of the study, involving identification and recognition of the salient findings and using them to arrive at a diagnosis or differential diagnosis (or a suggested pathway for further investigation). The second, equally important element is the communication of those findings and conclusions clearly, usefully and unequivocally in a report. Skill in one of these areas does not necessarily imply skill in the other [32].

While direct verbal discussion of findings occurs in some (especially more complex) cases, in most instances the dictated report represents the only opportunity for the radiologist to convey his/her interpretation, conclusions and advice to the referrer.

Failure to communicate relevant or urgent radiological findings (as opposed to failure to identify those findings in the first place) is a frequent cause of litigation [4, 24]. In 1997, failure of communication was the fourth most common primary allegation in malpractice suits against US radiologists; 60% of these claims resulted from failure to emphasise an urgent or unexpected abnormal finding [35]. A substantial part of our job is to ensure that referring physicians receive and understand our interpretations [4].

Increasingly, radiologists and radiology departments are expected to have robust methods in place to ensure that urgent findings are communicated safely to referring clinicians and that such communication is recorded and acknowledged [36]. Ten years ago a spokesperson for a major medical indemnity provider (while supporting the existence of a duty on the part of the radiologist to communicate reports in a timely and effective manner) said, *“I do not believe that there is an onus placed on the radiologist to pursue a reluctant clinician through the hospital corridors to ensure that he has received, understood and acted upon an abnormal report”* [35]. It is unlikely that the same latitude would be accepted by the courts today; the duty of care of a radiologist can no longer be thought to end with production of an accurate and timely report if that report is not effectively communicated [35].

Aside from the need for timely communication of reports, the capacity of a report to convey the thinking of a radiologist is variable. A radiologist’s understanding of the key messages included in a report and the interpretation of that report by the referrer can differ substantially [37]. It does not matter to a patient if a report contains all relevant findings if the report is not sufficiently clear for the referrer to understand what the radiologist means to say [38]. Poorly organised, poorly worded or error-strewn reports may contain all relevant information, but may not communicate it [1]. Incoherent, rambling or verbose reports may have the same effect [2]. From the patient’s perspective, the outcome is no better than if the report was filled with factual errors.

## Communication with patients

The audience for our reports has evolved from the referring clinician to now include patients, their families (and, sometimes, their legal representatives) [19]. Providing radiology reports (or key findings) directly to patients is controversial [35], but is likely to represent a growing future trend, with patient advocacy groups demanding the ability to directly consult with all medical providers, including radiologists [24]. Inappropriate or flippant language must be avoided. Radiology reports are communications between doctors, not directly between a radiologist and a patient, and should be factual, using correct and precise language. However, the language chosen should reflect the possibility that the patient may also be shown the report; some sensitivity is required.

In the USA, the Mammography Quality Standards Act (MQSA) mandates direct radiologist-patient communication in breast imaging [35], typically via the format of written standardised letters from radiology department clerical staff notifying patients of the clinical significance and follow-up recommendations in the report. Follow-up questions from the patient often lead to discussion with their primary care physician or (less frequently) written communication with the radiologist or the physician referrer. These further communications are usually based on the original written report rather than direct review of the source images. Thus, third parties can become conduits for information, which may not be clearly understood and which may become distorted.

## Images in reports

Radiology reports have traditionally been entirely textual, using word-based descriptions to convey meaning and intent, despite the fact that the meaning we attempt to convey is based upon images. Humans process and retain visual data much more effectively than words and text: images are deciphered in parallel and incorporated directly into long-term memory, while words are processed in sequence in short-term memory [41]. Many radiologists find it difficult to remember patients’ names, but are instantly able to recall their medical circumstances once shown images from their radiological studies. Surely, then, if we could find a means of incorporating images into reports, we could improve understanding and communication. This would have benefit for referrers and patients; in attempting to explain the nature of a radiological finding to a patient, it is much easier to show images of a mass than to try to describe it in words that may be unfamiliar to the patient [39]. This is one possible means of improving the perceived value of radiologists in patient care [40].

In recent years, incorporation of key images into textual reports has been advocated, with annotation of areas of interest using standardised symbols and/or free text [25, 41]. A shift from the printed word alone to image-centred structured reports, combining textual and image data, is suggested, although this has yet to be validated or supported by clinicians [2]. This seems intuitively a good idea; why, then, is it not already standard? The answer lies in the technology underpinning Radiology Information Systems (RISs), which store and transmit radiology reports. RISs, and the interfaces that transmit information from them to electronic medical records (EMRs), use older standards, such as Health Level 7(HL7), which cannot easily accommodate image data [3]. We await widespread availability of newer information technology platforms and standards that will facilitate multimedia reports.

## Impact of PACS/VR

In the era of analogue imaging and reporting, before PACS/RIS, generation and transcription of radiology reports were slower than today, often measured in days rather than hours or minutes. Many potentially rate-limiting steps in the process could influence the speed of report provision. A referring clinician who wanted urgent results was usually required to come to the radiology department and interact face-to-face with the relevant radiologist. Frequently, the only means by which a referrer could view images was by physically attending at the radiology department. One of the benefits of this model was the fostering of close working relationships between referring clinicians and radiologists, who could each develop an understanding of the specific needs and individual quirks of the other, thereby facilitating the process of communication [17, 24, 25].

This scenario has changed. With the widespread adoption of digital imaging systems, one unintended consequence has been the diminution in the frequency of direct one-to-one contact between referrers and radiologists. Radiology images are now available throughout the distribution network immediately after completion of the study, and report generation is correspondingly faster. The capability to deliver reports rapidly (turn-around time) has now become a measure of the quality of performance of a department, often without reference to report quality [24, 25]. Radiologists are under increasing pressure to deliver reports quickly, and this can translate into a decrease in report quality.

The older methods of report generation, involving transcriptionists, are often considered the gold standard against which the quality of the output of voice-recognition (VR) systems should be measured, but transcriptionists were also not perfect. One paper reported that 33.8% of reports required editing by radiologists prior to final approval after transcription, and in 5.6%, the changes were significant in terms of patient impact [42]. Furthermore, in the past it was acceptable to state in the literature that *“time pressures…preclude any but the most cursory inspection of reports by the dictating radiologist. I do not advocate a detailed reading of all one’s reports; it simply isn’t practical”* [16], although wiser authors always maintained that proofreading and correcting transcribed reports remained the radiologist’s responsibility [43].

It would be a mistake to believe that VR dictation can translate our thoughts and intent in an error-free manner. VR is based on mathematical algorithms that predict the likelihood of a word on the basis of the two previous words. They do not understand syntax or grammar and may produce nonsense. While the accuracy of radiology VR has been measured as being as high as 98%, this translates into 10 errors in a 500-word report (not an unlikely length in cross-sectional imaging), which may radically alter the sense of a report [3]. The use of VR software has been found to significantly increase the error rate relative to dictation and manual transcription [44], probably due to failure by radiologists to invest time and effort in identifying and correcting errors prior to final report validation.

A recent review of 378 reports originating from a single department in a single 24-h period found that 90 reports contained errors (mostly spelling and grammar, followed by missense and then nonsense). Seven errors were considered significant and nine very significant. Errors were more frequent in reports of more complex studies, and the error rate correlated with the length of the report (longer reports contained a higher rate of errors per report) [45].

In the analogue days, proofreading was often left to transcriptionists, who might have developed experience of the speech cadences and expressions used by individual radiologists. VR systems are attractive to business managers, as they recoup the initial cost of their adoption rapidly because of savings in transcription costs. However, the hidden costs of radiologists’ editing time is usually not included in the cost-benefit calculation [3]. It has been estimated that these tasks can increase the time required to report a given study by 20–30% over the time required for actual interpretation and initial dictation [46, 47]. This represents a significant contribution to radiologists’ workload, but is also an opportunity, which we can use to optimise the clarity and comprehensibility of our reports before we finalise them [1]. Proofreading and correction of errors are necessary parts of the reporting process and should always be performed by radiologists before finalising reports. A radiologist could be considered legally liable for patient harm resulting from an unchecked report containing significant errors [2].

## Teaching reporting

In the COVER and ROVER surveys, a majority in both groups (referring clinicians and radiologists) were convinced that learning to report needs to be taught in a structured way [32]. Nonetheless, by the end of the twentieth century, 98% of US radiology residents received no formal instruction in radiology reporting [3], and in 2004, a US study reported that residents received no more than 1 h of didactic instruction in radiology reporting per year [2]. It has been suggested that a major reason most trainees receive little or no formal instruction in dictating is that there is a lack of consensus about what constitutes a good report [9] (an experienced colleague of the author’s has an interesting teaching technique, which usefully illustrates the value of clear reporting description. He asks one trainee to describe clearly the findings on a study; other trainees, who cannot see the images being described, draw their understanding of what their colleague is reporting. This, better than any lecture about specific word usages, teaches the need to use clear language about what we wish to convey).

Despite the perceived need for formal training in reporting, most trainees acquire their own style of reporting by assimilating and adapting the styles of senior colleagues with whom they interact during training [2]. However, trainees are often unaware of the effectiveness and appropriateness of their style [18]. By contrast, radiographers who are empowered and trained to issue clinical reports in some jurisdictions are much more likely to receive formal training in reporting [19]. Editing by more senior colleagues of reports generated by radiology trainees has been shown to improve perceived report clarity, brevity, readability and quality, indicating that elements of reporting style can be usefully taught [2, 48].

## Structured reporting

The style and content of structured reports, and preferences for or against them, have changed over the years as the capability to embed templates within VR systems has evolved. In 1983, Friedman wrote that computerised reports might be more quickly put together by the radiologist, but were no easier read by the clinician if filled with trivia [8].

Over the years, standardisation of the language used in and the structure applied to structured reports has increased their popularity. In 2007, an American College of Radiology (ACR) Intersociety Conference concluded that

1. Structured reporting is the optimised reporting method, provided that structured reporting tools do not impede radiologist productivity
2. Reporting tools should enable a hybrid of speech recognition and structured reporting
3. Radiology professional organisations should create a repository of exemplary reports based on standard vocabulary [3].

Arising from this, reporting vendors created a new standard for exchange of radiology report templates under the standards of Integrating the Healthcare Enterprise (IHE), called Management of Radiology Report Templates (MRRT), which allows sharing and exchange of templates in the way DICOM allows many similar functions for images. RadLex terminology provides a single unified source of terms providing information in radiology reports [3]. For a given examination and clinical context, structured reports should list the same major elements in the same order, regardless of author [3].

Advocates of free-text reporting sometimes argue that the essential art inherent in the crafting of a prose report, involving structuring the body of a report to support a conclusion reached at the end of a reasoned description of findings, is lost in structured reporting. They may fear that removal of the need to follow a clear thought process to arrive at an ordering of observations reflecting their importance may interfere with the clarity of thought required to make sense of a complex study [3]. This might be a valid complaint if all radiologists composed their reports in clear, ordered prose. Sadly, the art of prose composition has decreased in importance in modern educational curricula, and today’s radiology trainees are probably less familiar with and capable of producing clear dictated prose reports than their forebears. Conversely, reports generated by trainees using templates effectively are probably more consistent and complete than ever before [3]. Not all radiologists are Hemingway, but most can fill in a form competently. While some of us might prefer to read Hemingway-esque prose in reports, we must settle for the homogenisation offered by structured reports that ensures no significant element is omitted in the absence of the widespread availability of the elegant prose composed by the few.

Communication of information (and diagnostic opinion) is key—how one arrives at that goal may vary (structured reports, bullet points, elegant language), but the destination remains the same.

Fundamentally, it has been suggested that narrative reports have evolved for the convenience of the report author, not the report reader [3]. Some radiologists “*confuse perfection of language with clarity of thought and communication*”. These are not synonyms and may compete with rather than complement one another, especially if the person reading the report does not share the linguistic views of the reporter [13].

Some radiologists may believe that their own crafted, nuanced narrative reports are better, clearer and easier to interpret than structured reports. The problem is that those reading their reports may not agree, and, if every radiologist entertains the same belief about their own reports, they cannot all be right. Structured reporting can eliminate this individuality and homogenise our output. Structured reporting retains some freedom for the would-be prose stylist—the conclusion. Once the body of the report has included all relevant findings and observations, the radiologist must summarise in a conclusion, and this remains an art of distillation of what is important and an elucidation of what the specifics of the structured report mean.

One other criticism levelled at structured reporting is that there is a risk that unexpected significant findings outside the clinician’s specific concerns may be missed by a referrer reading a structured report under time pressure, focusing only on that component of the report that matches the pre-test clinical expectation. Again, care in composing the conclusion of a structured report should minimise this risk, as long as the structured template used provides sufficient prominence for the conclusion [1].

A major additional advantage of structured report usage is the capability for mining reports for purposes of research and teaching, based on searches for specific terms or of specific report elements [3]. The use of Common Data Elements (CDEs) in reports greatly enhances this possibility. CDEs are "*data elements that are collected and stored uniformly across institutions and studies and are defined in a data dictionary*", essentially representing predefined questions and the sets of allowable answers to those questions. CDE usage, and its computer-readability, facilitates research based on radiology findings, including the prospect of including data from a much wider aggregate of institutions and reports than is usually currently possible [49].

When considering the stated preference of referrers for structured reports [31], radiologists may fear that adoption of structured reporting as standard may further the perceived diminution of our centrality in patient care, reducing our reports (which we view as clinical consultations among colleagues) to the status of laboratory reports. Halting or reversing this trend will not be achieved by insisting on free-text reporting unless we can guarantee that this will be of consistent high standard. Given that any form of writing, including radiology reporting, is inherently personal [3], we cannot give this guarantee. Better that we ensure that the information we provide is consistent, correct and accessible and devote our efforts to maintaining or enhancing our relevance by the quality of the conclusions we draw from findings listed in a structured manner.

The technical aspects of and the arguments for the widespread adoption of structured reporting are considered in greater detail in the recent position paper of the European Society of Radiology on Structured Reporting [50].

## Conclusion

Not all radiologists understand what their role is or make enough effort to approach a diagnostic problem with clear goals of extracting information from studies and formulating that information into a diagnosis or a plan to achieve what is needed diagnostically. Some think that a recitation of findings completes their responsibility; this can give radiology a bad name and can lead to other doctors thinking they can do our job better than we can, within their own specialty. Perhaps they can, if they understand the key clinical question and strive to answer it better than we do. To remain relevant, we have to think clinically and add value to the process of investigating and managing patients [40].

The principal value contribution arising from the work of radiologists is the radiology report, used as a means of conveying relevant information to referrers and of guiding further investigation and management. Traditionally, this has taken the form of prose reports of a widely variable standard. PACS/RIS and VR have greatly altered the way we do our work, speeding up generation of reports and their communication to referrers, but also diminishing the need for direct contact between referrers and radiologists. To maintain our relevance in patient care, we must put renewed effort into optimising the quality of our reports. These technological developments also provide an opportunity to do this by facilitating widespread adoption of structured reporting. Those among us who enjoy the exercise of crafting careful prose reports can still find an opportunity for this activity in writing report conclusions. Those for whom the manipulation of language holds no interest can rely upon report templates to allow them concentrate upon their interpretive skills. Those for whom clear communication does not come easily can be guided by the structure of templates to ensure their thoughts are communicated with clarity.

As recently endorsed by the European Society of Radiology [50], the future of radiology reporting lies in structured reporting. We should not fear it; it will not turn radiologists into machines spitting out mechanical data (the HAL of the title). It will not make any of us worse radiologists, it will make many of us better, and it will homogenise our work output in a positive way.
